# Role of Oxidative Stress in Varicocele

**DOI:** 10.3389/fgene.2022.850114

**Published:** 2022-03-23

**Authors:** Kaixian Wang, Yuanyuan Gao, Chen Wang, Meng Liang, Yaping Liao, Ke Hu

**Affiliations:** School of Life Science, Bengbu Medical College, Bengbu, China

**Keywords:** varicocele, oxidative stress, antioxidant, infertility, DNA oxidation damage

## Abstract

According to the official statistics of the World Health Organization, at least 48 million couples and 186 million people suffer from infertility. Varicocele has been recognized as the leading cause of male infertility and can affect spermatogenesis and cause testicular and epididymal disorders through multiple diverse pathophysiological processes. Reactive oxygen species (ROS) produced by oxidative stress have been reconciled as an important pathogenic factor throughout the course of varicocele. Testis respond to heat stress, hypoxia, and inflammation at the cost of producing excessive ROS. High levels of ROS can lead to infertility not only through lipid peroxidation or DNA damage, but also by inactivating enzymes and proteins in spermatogenesis. This review studies the oxidative stress and its role in the pathophysiology and molecular biology of varicocele in the context of a decline in fertility.

## Introduction

**FIGURE 1:**
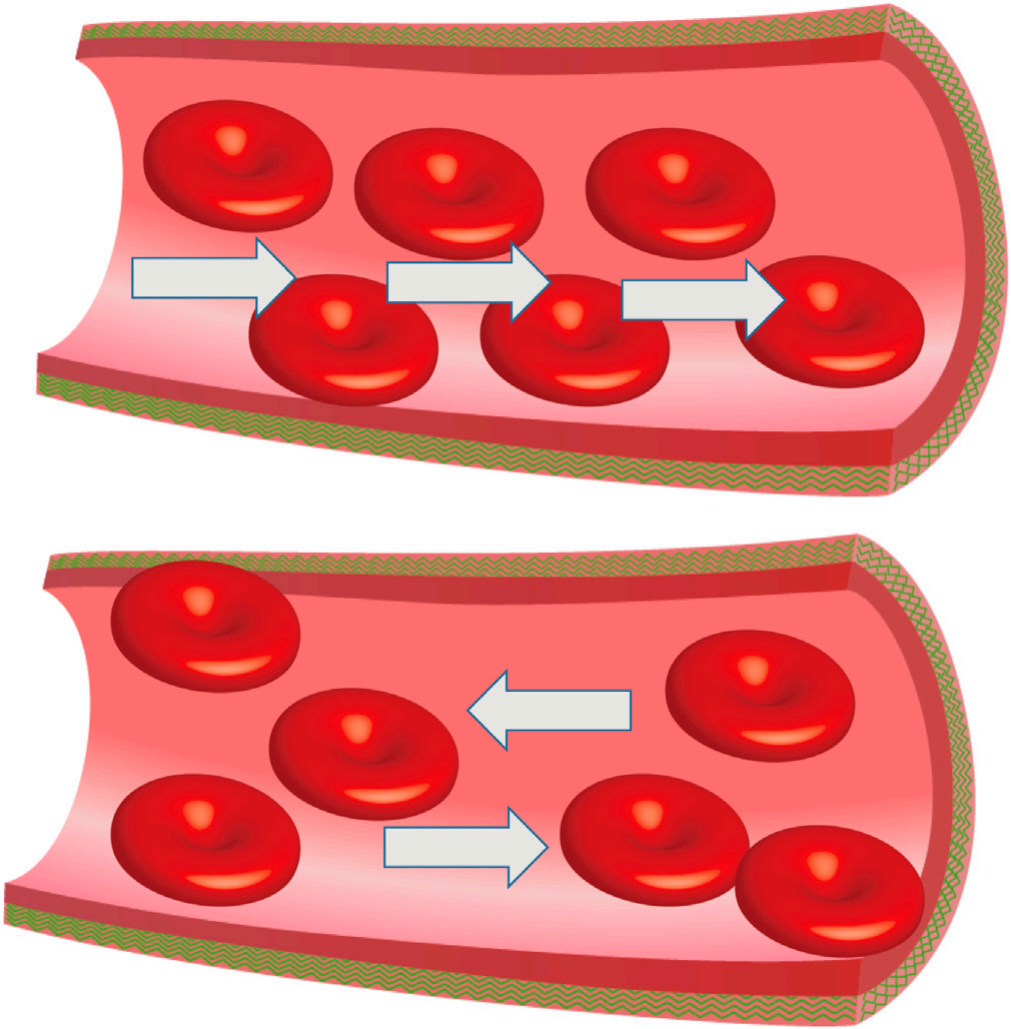
Abnormal venous valve function. Damaged venous valves result in local venous return, the white arrow represents the direction of blood flow.

**FIGURE 2:**
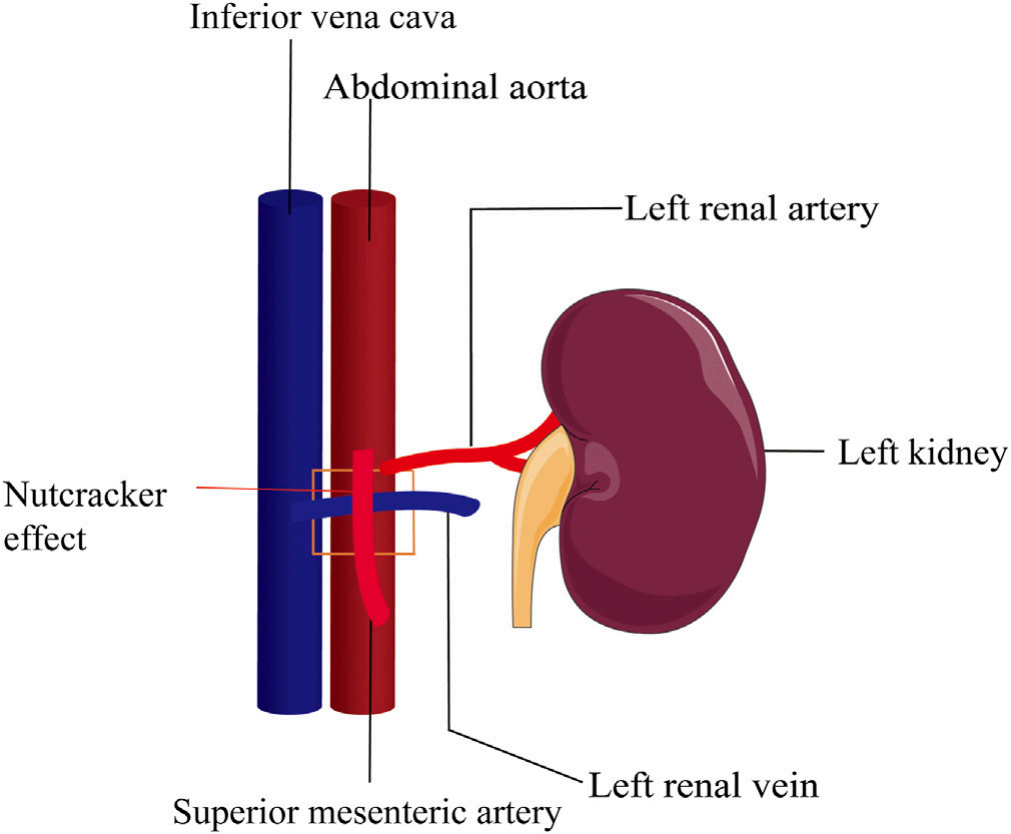
Nutcracker effect. Compression of the superior mesenteric artery on the left renal vein leads to elevated renal vein pressure and spermatic vein return disturbance. The square area represents the superior mesenteric artery pressing against the renal vein.

Infertility affects 13–15% of couples which causes widespread concern around the world and malefactors are directly or indirectly responsible for about 60% of infertile couples (Thonneau et al., 1991). The term “varicocele” was introduced by Curling in 1846 (Curling 1846) (Table 1) and it has been reported that the occurrence rate is between 15 and 20% in the general population and 30–40% in infertile men (Practice Committee of the American Society for Reproductive Medicine, 2014). Men with varicocele have a lower total sperm count, testosterone levels, and reduced testicular size on the same side of the varicose vessels compared to those without varicocele. There are three etiologies that can cause varicocele to occur: 1) loss or dysfunction of venous valves results in local venous return (Figure 1), 2) the angle difference between the left and right testicular veins and the left renal vein and vena cava, and 3) the superior mesenteric artery compression of the renal vein leads to a “nutcracker effect” (Figure 2) of elevated venous pressure in the spermatic vein return resulting in venous obstruction.

**TABLE 1:** The history of the development of varicocele.

Study	Results
Ambroise et al.(Ludwig 2022) (1600's)	Varicocele was defined
Curling et al.(Curling 1846) (1846)	The term “varicocele” was proposed
Barfield et al.(Scott and Young 1962) (1880)	Varicocele can cause infertility
Macomber et al.(Macomber and Sanders 1929) (1929)	Fertility was restored by bilateral spermatic vein ligation
Tullocb et al.(Tulloch 1952) (1952)	Fertility was restored after surgery in a case of azoospermia with varicocele
Stephenson et al.(Stephenson and O'Shaughnessy 1968) (1968)	Relationship between Hypospermia and scrotal temperature in patients with varicocele
Zorgniotti et al.(Zorgniotti and Macleod 1973) (1973)	The scrotal temperature was significantly increased in patients with varicocele
Alkan et al.(Alkan et al., 1997) (1997)	Spermatic vein resection can reduce oxidative stress
Turner et al.(Turner 2001) (2001)	Classic varicocele animal model
Lee et al.(Lee et al., 2006) (2006)	ROS caused by hypoxia aggravates testicular tissue damage in patients with varicocele
Wang et al.(Wang et al., 2010) (2010)	Testicular hypoxia is an important pathophysiological feature of varicocele patients
Agarwal et al.(Agarwal et al., 2012) (2012)	Oxidative stress is a major contributor to the pathophysiology of varicocele
Choi et al.(Choi and Kim 2013) (2013)	Relationship between ROS and sperm damage in patients with varicocele
Ziliang et al.(Ji et al., 2014) (2014)	microRNA and varicocele
Zhao et al.(Zhao et al., 2018) (2018)	Oxidative stress-related LNC RNA GADD7 in spermatozoa of infertile men with varicocele

Spermatogenesis is the process by which diploid gamete cells produce haploid sperm. During spermatogenesis, diploid male primordial germ cells develop through meiosis and differentiation into haploid sperm, which pass genetic material to the next generation. It is regulated by a complex network of interactions between various testicular cells (Mclaren 2012). Mesenchymal cells regulate and support the development of germ cells by producing androgens and secreting various factors by Sertoli cells and peritubular myoid cells. Spermatogenic cells can program the response of target cells to androgens and regulate spermatogenesis (Henriksen et al., 2020). Changes in the microenvironment, such as heat stress and hypoxia, may affect a certain process of spermatogenesis and cause a decline in male fertility.

Only about 20% of varicocele patients are infertile (Green et al., 1984), but it accounts for about 19–41% of patients with primary infertility and approximately 80% of secondary male-caused infertility (Shalgi et al., 2013). Decades of studies have shown that semen oxidative stress and sperm damage are major contributors to varicose vene mediated male infertility with a long course of illness (Cho et al., 2016) and shows that varicocele is a progressive disease rather than static damage, meaning that it will not disappear naturally (Selvam et al., 2020).

## The Effects of Reactive Oxygen Species

ROS widely refers to free radicals and non-free radicals derived from oxygen, including superoxide anion, hydrogen peroxide, hydroxyl radical, ozone and singlet oxygen, which have high chemical reactivity because of unpaired electrons. The abnormal sperm morphology, lipid peroxidation, DNA fragmentation, and protamine scarce in men with varicocele were apparently higher than those in fertile individuals (Talebi-Yazdabadi et al., 2021).

### DNA Oxidative Damage

Sperm DNA integrity is essential for the birth of healthy offspring (Krawetz 2005) and the imbalance between excess ROS production and antioxidant protection leads to changes in nuclear and mitochondrial sperm DNA, resulting in reduced fertility in men with varicocele (Roque and Esteves 2018). Excessive oxidative stress can influence sperm telomere length and sperm DNA integrity, the shortening of telomere length in sperm and white blood cells may explain the increasing DNA fragments in sperm (Tahamtan et al., 2019). Sperm chromatin has a large number of alkali-unstable sites, which are mainly located at repeated DNA sequences and are prone to DNA twisting during chromatin packaging. Plenty of evidence suggests that sperm DNA fragmentation (SDF) can be regarded as a biomarker of chromatin impairment and takes an indispensable and significant effect in male infertility and reproductive success (Esteves et al., 2021). Couples with higher SDF in male partners had longer pregnancies among couples who conceived independently. SDF can be used as an index to detect sperm chromosome damage in semen, and may be related to varicocele, male accessory gland infections, inadequate lifestyle, and gonotoxin exposure. It is also associated with intrauterine insemination and assisted reproductive technology (ART) outcomes (Avendaño et al., 2010).

**FIGURE 3:**
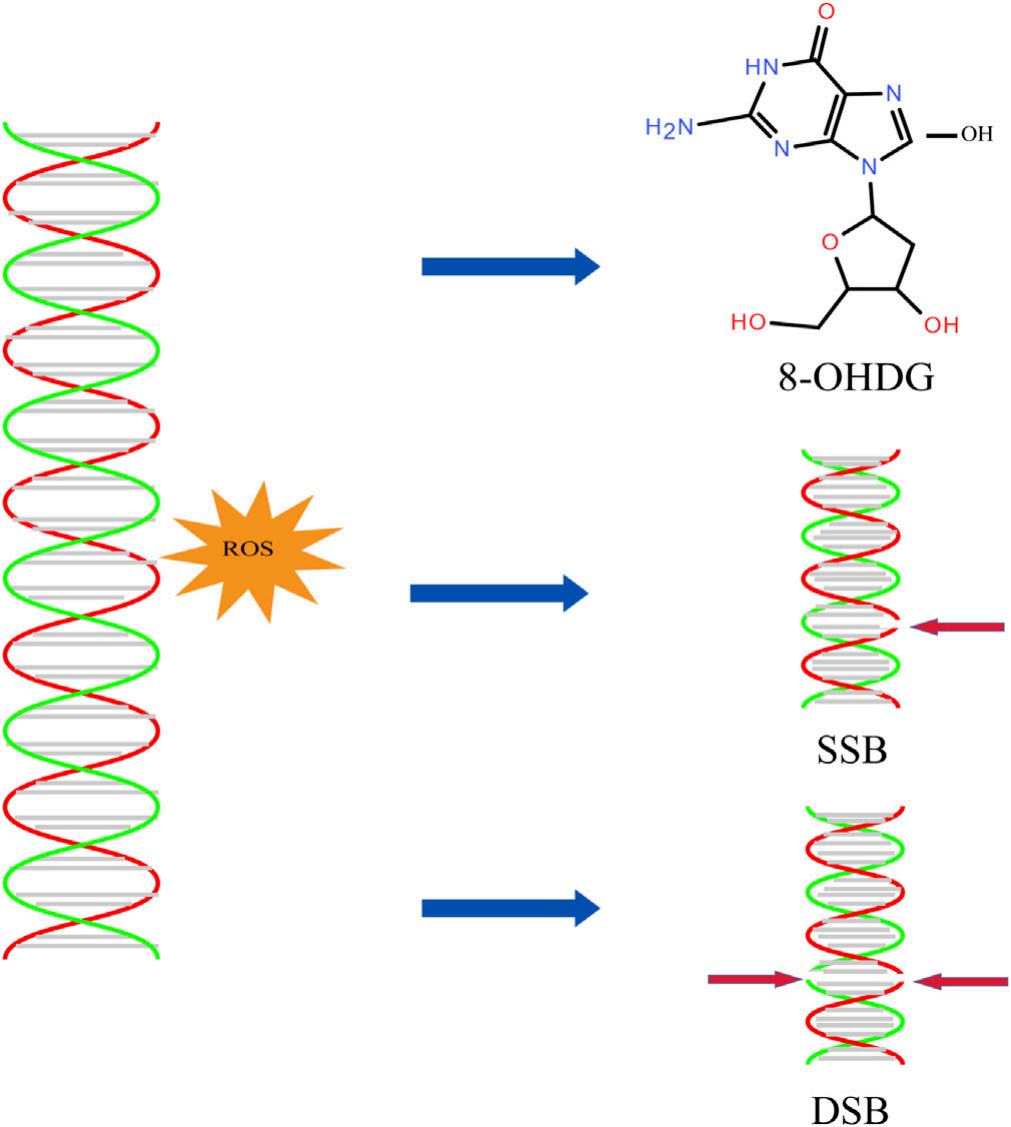
DNA oxidative damage. ROS can induce the production of 8-OHDG and the breakage of single stranded DNA and double stranded DNA.

Studies have shown that low-fertility bulls have four times the SDF of high-fertility bulls. The effect of SDF on reproductive success depends on the balance between the degree of DNA damage and the ability to repair DNA. Although the repair process may occur at the prokaryotic stage before the ligand, it has been hypothesized that sperm DNA damage exceeds the repair capacity of oocytes or that oocytes are unable to repair DNA damage, thus affecting the developmental potential of embryos and the health of offspring (Hsu et al., 2006). The guanine base (G) is the most common organic DNA base, which is subject to oxidative attack by free radicals and is converted to 8-hydroxyguanine (8-OHG) (Figure 3). By investigating the DNA of sperm of infertile men and comparing it with fertile individuals, it found that the 8-OHG in semen of infertile men was about 100 times higher than sperm of fertile men (Noblanc et al., 2012). Excessive ROS overwhelms the ability of sperm defenses against oxidative stress and causes damage sperm DNA, resulting in point mutations, deletions, chromosomal rearrangements, and single-stranded or double-stranded DNA breaks. The DNA fragment rate of varicocele patients was 32.4%, 2.6 times higher than that of men in the normal reproductive period. Studies have reported that chromosomal abnormalities and microdeletions in the Y chromosome were associated with varicocele and may cause azoospermia in their offspring (Moro et al., 2000). Among the studies, 19.3% patients had autosomal changes, including inversions of chromosomes 9 and 2, translocations between chromosomes 4 and 15, deletions of chromosome 4, and insertions of chromosome 9 (Rao et al., 2004). The rate of sperm DNA methylation in varicocele patients was also lower than that in normal reproductive population, but the difference disappears after varicocele surgery.

The role of excessive ROS levels can also lead to mitochondrial DNA (mtDNA) damage (Bui et al., 2018). Mitochondrial dysfunction has been considered as one of the main factors affecting the normal physiological function of sperm, ROS produced by it can cause mitochondrial damage in turn (Panner Selvam et al., 2021). It has been found that up to 81.7% of varicocele patients had 4,977 base pairs missing in sperm mitochondrial DNA, in contrast, only 15.5% of the control population had missing pairs (Spiropoulos, 2002). ROS can directly damage mtDNA by damaging mitochondrial intima and at the same time lead to electron leakage in the electron transport chain, thereby increasing oxidative stress production (Barati et al., 2019). Mitochondria have been thought to be more prone to attacks by ROS due to the absence of material damage mechanisms and histone protective mechanisms. The mutation rate of mtDNA is regard as much as 100 times that of nuclear DNA. There is evidence that oxidative damage of mtDNA can trigger the development of ischemia-reperfusion injury and may lead to cellular dysfunction and tissue damage (Noblanc et al., 2012).

The decline of mtDNA copy number aloneis sufficient to induce myocardial apoptosis, and the change of copy number of mtDNA were consistent with changes in antioxidant capacity. mtDNA damage or depletion affects the respiratory chain, enhances oxidative stress and inflammatory response, and induces apoptosis. Once oxidative damage occurs to mtDNA and the key encoding protein oxphos is lost, it leads to more ROS production and mitochondrial destruction. Studies show that oxidative stress caused by sperm incubation with hydrogen peroxide leads to severe loss of motility and DNA damage (Blumer et al., 2011). NLRC4, as an inflammatory factor, it plays a crucial role in innate immune responses to various pathogenic organisms, tissue damage and other cellular stresses. Inhibition of mitochondrial ROS release or degradation of intracellular mitochondrial DNA can eliminate NLRC4 inflammosome activation.

### Oxidative Protein Damage

**FIGURE 4:**
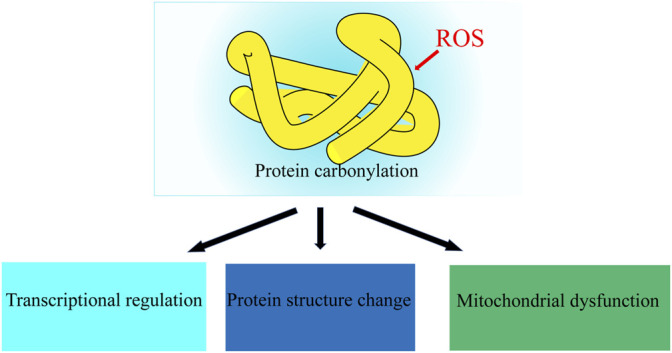
Oxidative Protein Damage. ROS leads to abnormal protein structure and function.

Proteins are prime targets for free radicals and other oxidants in both the intracellular and extracellular environments and can be divided into the main chain and side chain oxidation. The main effects of ROS on proteins are modification of amino acid residues, cleavage of peptide bonds, structural, conformation changes, and protein cross-linking polymerization (Behrendt and Ganz 2007). The main sites of free radical attacks are aromatic or heterocyclic rings of amino acid residues, resulting in oxidation or breakage of rings, forming different oxidation products (Figure 4), which can cause various damage to the protein components of cells, leading to denaturation, misfolding and aggregation, and even apoptosis (Agarwal et al., 2012, Hamada and Esteves). Due to the presence of multiple proteins in biological systems and the potential hydrolytic repair of proteasome, fragments produced by main chain breakage can hardly be used as markers of oxidative damage to proteins. Molecular pathways related to mitochondrial function, free radical scavenging, protein ubiquitination, and post-translational modifications (PTM) were all defective in the sperm of unilateral varicocele (Agarwal et al., 2015). In bilateral varicocele, the expression patterns of sperm proteins APOA1, TOM22, and TGM4, which are associated with oxidative stress and SDF are also altered (Agarwal et al., 2016).

Proteins may be a highly sensitive indicator of chronic oxidative damage in mammals because some proteins have long half-lives and are prone to be damaged due to the accumulation of OS. Oxidative damage of proteins caused by ROS can associate with aging, tumors, diabetes, and many neurodegenerative diseases (Butterfield et al., 2014). Protein carbonylation and tyrosine nitration can be regarded as markers of oxidative damage of proteins. The change of carbonylation level and nitro modified SOD *in vivo* can reflect the degree of oxidative damage of proteins and may be used as markers to detect varicocele.

Oxidative stress can affect the epidermal epithelial binding protein ZO-1 and epidermal function in varicocele model rats (Guang-Wei et al., 2019). The increase of ROS may lead to the changes of sperm phosphorylation, acetylation and ubiquitin modification, which may lead to the abnormality of spermatogenesis or sperm function. Damage to the proteasome complex may lead to accumulation of misfolded proteins that add sperm DNA damage and apoptosis in patients with bilateral varicocele. Abnormal expression of proteins involved in the acetylation process may trigger the p53 transcription factor, which in turn activates the apoptosis process of defective sperms in patients with varicocele (Nazmi et al., 2012). Oxidative damage to proteins such as SDHA in mitochondria may also affect the tricarboxylic acid cycle (TCA), electron transport chain, and energy metabolism (Selvam et al., 2020).

### Lipid Peroxidation

**FIGURE 5:**
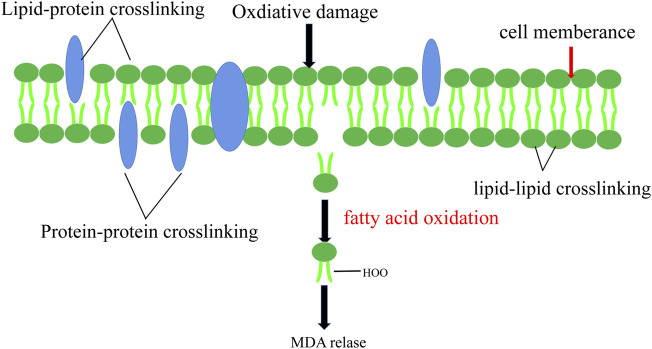
Lipid Peroxidation. Lipid peroxidation leads to the change of cell membrane structure and the release of MDA.

Sperm are particularly vulnerable to ROS damage due to their plasma membranes being filled with polyunsaturated fatty acids (PUFA) with multiple double bonds. PUFAs suffer an initial electrophilic attack by ROS which will eventually lead to the formation of malondialdehyde (MDA) (Aitken 2017). Induction of lipid peroxidation cascades is the result of reduced sperm function caused by ROS levels exceeding in sperm. Extensive lipid peroxidation alters the accumulation, structure, and dynamics of lipid membranes, excessive MDA production due to LPO can react with amino compounds such as protein, nucleic acid and cerebral phospholipin to cross-link them (Figure 5). (Gaschler and Stockwell 2017).

Sperm motility declines due to the lack of adenosine triphosphate (ATP) which is caused by lipid peroxidation. Oxidative stress has a knock-on effect of the hypothalamic axis and can disrupt the secretion of sex hormones. ROS reduces male sex hormone levels and disrupts the reproductive system (Doshi and Khullar, 1996). In patients with varicocele, the content of LPO in testicular tissue increases significantly, and this high concentration of LPO damages testicular spermatogenic cells and sub-cell membranes, resulting in spermatogenic dysfunction. The content of MDA is increased with the severity of varicocele, suggesting that there is a mechanism for the increase of MDA with varicocele (Shiraishi et al., 2012). Loss of membrane integrity leads to increased permeability and a loss of the ability to regulate intracellular ion concentrations involved in controlling sperm motility (Blumer, Restelli, Giudice, Soler and Cedenho 2011). Hassan showed that the percentage of lipid peroxidation in varicocele rats was significantly higher than in the control group (Hassani‐Bafrani et al., 2019a). However, the effect of lipid peroxidation on varicocele patients’ reproduction needs more research.

## Mechanisms of Oxidative Stress in Varicocele

Oxidative stress has been considered as one of the most important causes of poor fecundity in varicocele patients. The decrease in seminal plasma total antioxidant capacity (TAC) is associated with impaired semen parameters. However, the relationship between TAC and semen parameters in infertile varicocele patients requires further investigation (Wang et al., 2009). Low doses of oxidative stress are necessary for spermatogenesis, the condensation of sperm nuclear chromatin during spermatogenesis is achieved by the formation of ROS-induced disulfide bonds between cysteine residues in protamine’s. Local testicular heat stress, hypoxia, and inflammation caused by varicocele can increase the production of ROS in tissues underunder conditions of venous blood reflux, abnormal heat exchange. Excess oxidative stress in the body can lead to sperm damage, sperm capacitation, hyperactivation, acrosomal reaction, and sperm–ovule fertilization. Oxidative stress and poor chromatin packaging can affect the integrity of sperm chromatin and lead to male infertility (Lakpour et al., 2008). In the case of several pathogenic factors of varicocele, they will lead to the breaking of local oxidative balance, induce oxidative stress, and produce excessive ROS.

### Testicular Temperature and Oxidative Stress

Grma first reported that increased scrotal temperature in patients might be one of the reasons for infertility in 1921 (Olson and Stone 1949). This was first reported by the British surgeons, S. Brown, proposing that varicocele can cause male infertility (Kursh 1987) and J. I. ALI finding that the temperature of the left scrotum was significantly higher in infertile men with varicocele compared to normal males because of poor venous return in the testis and spermatogenesis being a temperature-sensitive process (Mieusset and Bujan 2010). *In vitro* and *in vivo*, studies have shown a direct temperature-dependent relationship between heat exposure and ROS production (Alvarez and Storey 1985). The optimum temperature for spermatogenesis is 2.5°C lower than that of the core body (Mariotti et al., 2011). Cells in the testicular tissue began to undergo apoptosis leading to the spermatogenic function being weakened, and the quality and quantity of sperm decreasing with only a 1–1.8°C increase in scrotal temperature (Jung and Schuppe 2010). Varicocele has been considered as a chronic genital heat stress condition. The scrotal temperature of varicocele patients is about 1.5°C higher than that of healthy people (Shiraishi et al., 2009). There is a significant association between oxidative stress and varicocele-related infertility caused by testicular heat therapy, which can lead to low sperm function.

**FIGURE 6:**
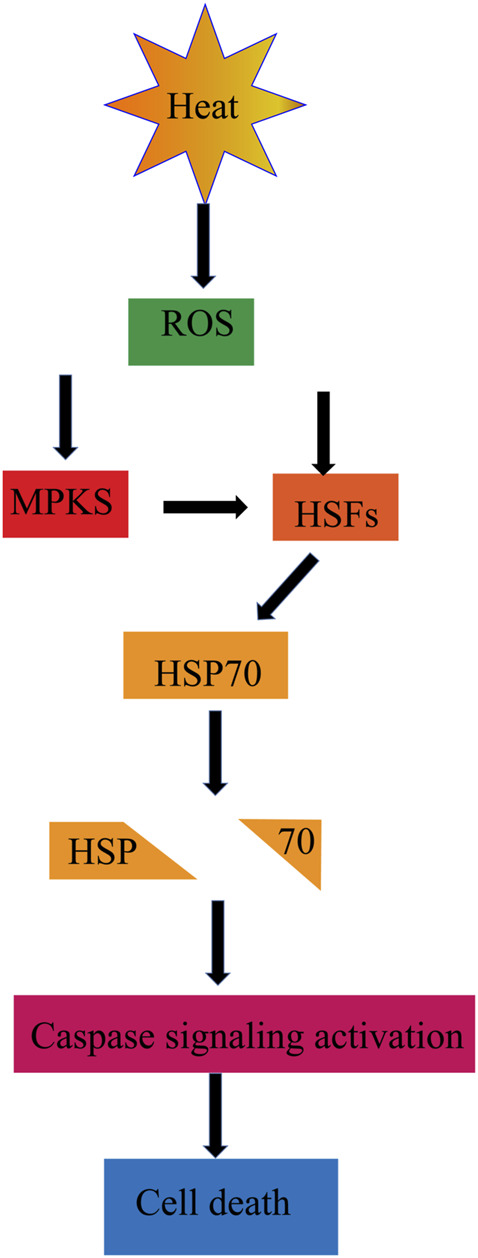
ROS and HSPs. ROS produced by heat stress leads to changes in HSP related and pathways.

Heat shock proteins (HSP), as a broad category of housekeeping proteins, are constitutively expressed in cells to regulate various cellular pathways like transport, translation, transcription, and signal transduction. HSP associated proteins can significantly alter (Figure 6) in response to stimuli of heat stress (Parsell and Lindquist, 1993). The inability to produce adequate concentrations of functional HSP may explain the increased sperm protein denaturation, apoptosis, and male infertility in varicocele patients. HSPA2 mRNA and protein expression levels were lower in oligozoospermic men with varicocele, but HSPA2 protein activity increased after varicocele resection. Studies show that the increase of heat shock factor 1, a major transcription factor with apoptosis-inducing effects, regulates the expression of HSP. Heat exposure speeds up cellular metabolism and energy expenditure both *in vitro* and *in vivo* experiments. Temperature rise in the range of 34–40° induces oxidative stress in tissues and increase the MDA level of sperm in mice and rabbits (Mikhael et al., 2018).

Heat stress also can induce the production of NOS. Studies have shown that the testicular NOS content in patients with grade II and III varicocele is significantly higher than that in patients with grade I varicocele and higher than that in normal people. When the spermatic vein is abnormally dilated, blood from the scrotum to the groin can backflow and deposit, blocking the heat exchange established between the testicular arteries and veins (Alsaikhan et al., 2016). NO is easily diffused to the membranes of various germ cells and can react with superoxide anions produced in the mitochondria of germ cells to produce active metabolites such as peroxynitrite and peroxynitrous acid. NOS inhibitor therapy can alleviate testicular atrophy and improve spermatogenesis in cryptorchidic mice induced by Hoxa 11 knockout (Defoor et al., 2004).

### Hypoxia and Oxidative Stress

**FIGURE 7:**
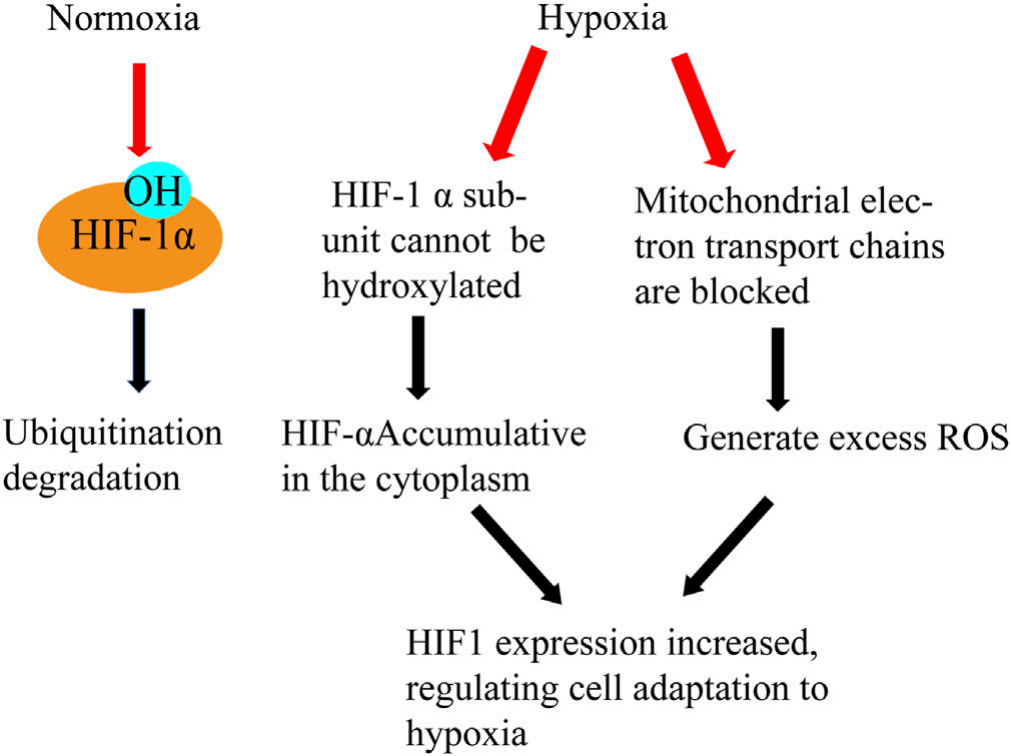
Hypoxia and HIF. Effects of normoxia and hypoxia on HIF- α Different processing results in different functions of HIF and the generation of ROS.

Hypoxia is one of the most important factors of male infertility caused by varicocele (Wang et al., 2009). Under the condition of hypoxia, mitochondria will produce a large amount of ROS, and excess ROS will further induce the body to produce hypoxia stress response. In patients with varicocele, hypoxia can reduce testis tissue oxygen partial pressure and cause a metabolic disorder (Gat et al., 2010). With the occurrence of hypoxia in the testis microenvironment, the expression of a series of hypoxia-related factors and related genes changed, which had an effect on the testis microenvironment again. Hypoxia-inducible factor-1 (HIF-1) is the specific factor produced when the tissue hypoxia, expressed in germ cells and binds to vascular endothelial growth factor (VEGF)and plays a leading role in alleviating the damage caused by tissue hypoxia (Figure 7). Studies have shown that hypoxia can increase the expression of HIF-1α in the testis of rats with varicocele, and the apoptosis of spermatogenic cells is significantly increased. The degradation pathway of HIF-1α is blocked after hypoxia occurs, and HIF-1α accumulates and enters the nucleus (Zhang et al., 2016).

Studies have shown that when the CRISPR/Cas9 gene editing technique is applied to silence the HIF-1α gene of varicocele rat testis, HIF-1α regulates the spermatogenesis in fertilized varicocele rats and the PI3K/Akt signaling pathway plays a regulatory role in this process. Active HIF-1 binds to a variety of hypoxic-sensitive genes and promotes transcription of these target genes, including erythropoietin (EPO) and vascular endothelial growth factor (VEGF). VEGF is a 21 kDa glycoprotein regulated by HIF-1 and has a specific mitotic effect on vascular endothelial cells which can regulate endothelial cell proliferation, angiogenesis, and vascular permeability. The expression of VEGF in testis of varicocele rats is increased, and recent studies have shown that intratesticular injection of VEGF can improve spermatogenesis and reduce apoptosis (Tek et al., 2009).

Prodynamin 2 (PK2), a multifunctional protein, interacts with vascular endothelial growth factor and other factors to promote testicular growth and maintenance function. The expression of PK2 mRNA is induced during hypoxia, mainly at the primary spermatocyte stage and increased in varicocele rats. The increase of PK2 can lead to the increase of intracellular calcium ions, leading to endoplasmic reticulum stress and apoptosis (Li et al., 2020). Apoptosis of spermatogenic cells was observed in the varicocele rat model when the expression of p70S6K and P-P70S6K in the nucleus and cytoplasm of spermatogenic cells was significantly increased and the expression of p-Akt and p-p70S6K decreased after HIF-1α gene silencing (Zhang et al., 2013). The combination of VEGF and VEGFR2 (phosphorylation) activates the PI3K/Akt pathway leading to the expression of P-Akt and PP70S6K proteins in the testis of varicocele rats (Wang et al., 2021).

### Inflammation and Oxidative Stress

Inflammation can be associated with multiple types of reproductive disorders, such as azoospermia, and may lead to infertility (Flannagan et al., 2019). Varicocele has been proven to be a chronic inflammatory vascular disease which can lead to local chronic inflammatory response. Oxidative stress leads to the damage of biomolecules and causes the body to produce endogenous damage related molecular patterns and cytokine release, activate the signal pathway downstream of PPRs, recruit and activate more inflammatory cells, and cause chronic aseptic inflammatory response in the body system. Some studies have confirmed that proteins involved in inflammatory pathways are differentially expressed in patients with varicocele. Elevated levels of pro-inflammatory cytokines have been observed in the seminal fluid of patients with varicocele (Zeinali et al., 2017). In varicocele patients, the increase of pro-inflammatory factors leads to the decrease of skeletal protein secreted by supporting cells. This results in the increase of blood testosterone barrier permeability, and ultimately the destruction of immune isolation. Sperm antibody repair tries to make a large amount of sperm cohesion, thus reducing the motility of sperm.

Bonyadi found that sperm antibody concentration in semen increases and sperm motility decreases significantly by detecting semen from patients with varicocele. Neutrophil products can be potential diagnostic biomarkers and therapeutic targets for varicocele-caused subfertility (Nazari et al., 2017). The link between hypoxia and inflammation is also closely related and inflammation can lead to local or systemic hypoxia, such as an abscess. Severe local hypoxia due to inadequate blood supply, or acute pulmonary inflammation, was found to impair gas exchange. The incidence of epididymal inflammatory masses was significantly higher in patients with varicocele and in patients with severe varicocele (Vivas-Acevedo et al., 2014). The link between inflammatory hypoxia and inflammatory signals works both ways, hypoxia also exacerbates inflammation by activating inflammatory pathways and affecting the fate and function of immune cells.

**FIGURE 8:**
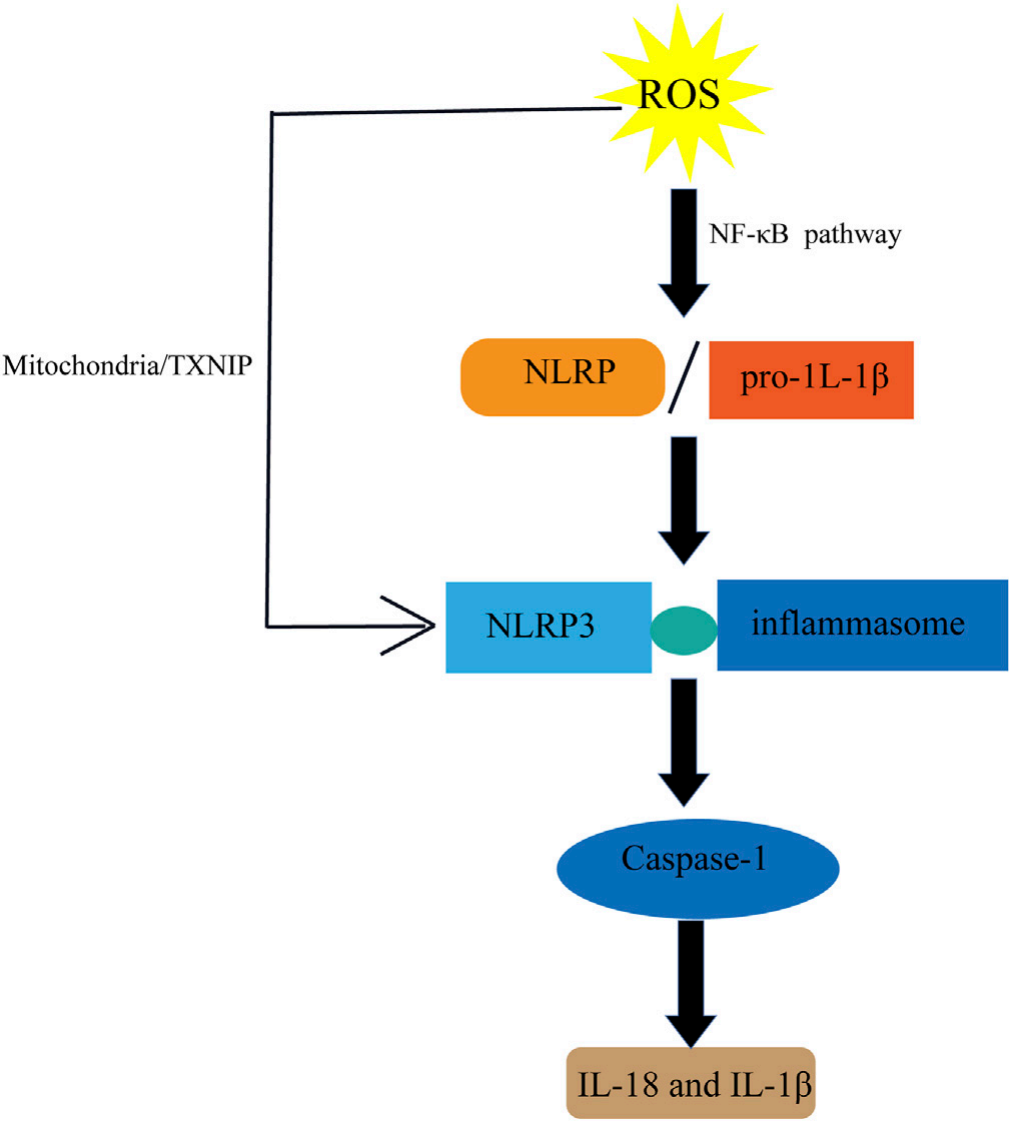
Inflammation and ROS. ROS induces the activation of NLRP pathway to produce IL-18 and IL-1 β.

Varicocele stimulates the release of pro-inflammatory and inflammatory cytokines such as interleukin-1 (IL-1), IL-6, IL-8, and tumor necrosis factor (TNF-α) (Habibi et al., 2015). ROS may play a significant role in the NLRpyrin domain, containing activation of the NLRP3 inflammasome (Figure 8) that inhibits ROS blockade by chemical scavengers (Schroder and Tschopp, 2010). Inflammation also can lead to an increase in iNOS expression. Varicose vein excision can reduce caspase-1, IL-18, and IL-1β levels by analyzing the semen composition of patients 6 months before and 6 months after surgery. Thus, varicocelectomy improved sperm morphology and reduced inflammatory activity in the seminal plasma at 6 months postoperatively varicocele (Ata-Abadi et al., 2020).

## Experimental Antioxidant Therapy With Varicocele

**FIGURE 9:**
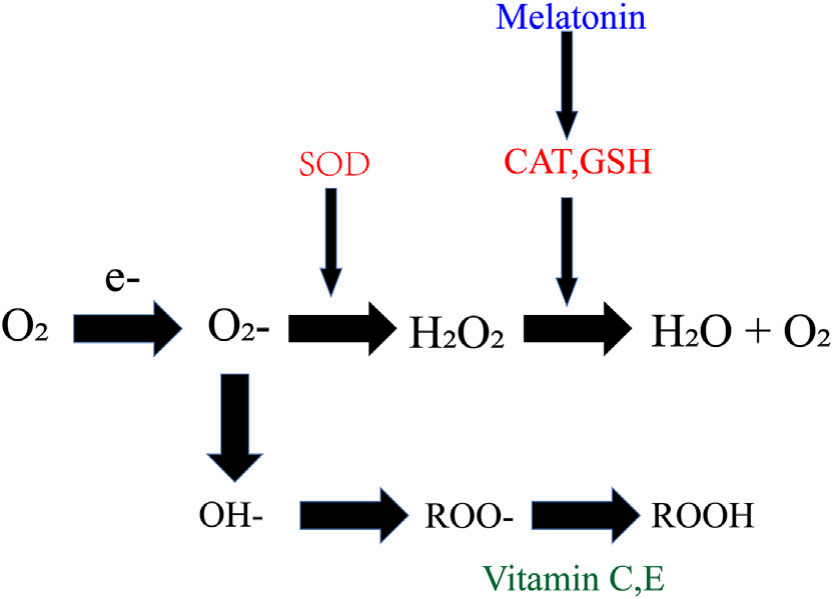
Antioxidants and ROS. Antioxidant function of vitamins C, E and melatonin.

A large number of clinical and animal model experiments have confirmed that varicocele can induce a series of pathological and physiological changes in testis, ultimately affecting the spermatogenic function of testis. Abnormal cell apoptosis and oxidative stress may play a major role in testicular injury, although the redox balance in the human body isstable, the local oxidative balance in the testis of varicocele patients is often disturbed. Some researchers have suggested that taking antioxidants, including vitamins for antioxidant purposes, selenium, and other bioactive substances can (Figure 9) reduce the extent of sperm DNA damage (Oliva et al., 2009). In patients with varicocele, the activity of redox related enzymes are reduced in the testis and epididymis, leading to a redox imbalance and cause testicular tissue damage and decreased sperm motility. Melatonin, vitamin C, and other bioactive compounds have been used to alleviate damage to testicular function caused by varicocele in animal modelsand research shows that Oral antioxidant treatment partly improves integrity of human sperm DNA in infertile grade I varicocele patients.

### Vitamins

Vitamins E and C are well-documented antioxidants that have been shown to inhibit free radical induced testicular sensitive cell membrane damage and reduce MDA estimates of lipid peroxidation in tissues. Vitamin C has been considered the most critical antioxidant in semen which accounts for 65% of the antioxidant capacity (Song et al., 2010). Studies have shown that a daily intake of 200–1,000 mg of vitamin C can significantly improve sperm quality, but according to a recent study, 28% of urban men and 55% of rural men had vitamin C intakes below the recommended daily allowance (Malekshah et al., 2010). Motility and morphology were significantly better in the vitamin C group than in the placebo group for varicocele rats (Cyrus et al., 2015). Vitamin C improves motility and morphology instead of sperm count after varicocele surgery in patients (Doshi and Khullar, 1996), but excessive doses of vitamin C also can make sperm DNA more vulnerable to damage from the oxidation of purine and pyrimidine bases.

Evidence from observational studies suggests that the combined use of vitamin B and vitamin E can improve sperm caused by varicocele. he single injection of vitamin B can significantly improve testicular volume and effectively reduce the ratio of sperm DNA fragmentation in varicocele rats at 4 months, and the ratio of LPO and DNA damage in varicocele rats was decreased after a vitamin E injection, but the ratio of sperm residual histone was no significant difference between the control group and Vitamin E group (Hassani‐Bafrani et al., 2019b). Vitamin E has also been shown to significantly reduce semen ROS levels in rat models (Khosravanian et al., 2014). The favorable association between vitamin E intake and serum levels of proinflammatory cytokines (Arablou et al., 2019). Oral doses of antioxidant vitamins seem to be a good option for improving fertility in patients with varicocele and enhances success rate in recovery.

### Melatonin

Melatonin, a potent free radical scavenger, is one of the hormones secreted by the pineal gland which can mitigate the reproductive effects of oxidative stress. Its treatment of varicocele rats showed antioxidant effects, improved reproductive hormone axis, CatSper expression, and fertility parameters. Endogenously elevated melatonin levels were relevant to decrease apoptosis of the Leydig cell, increase testosterone production, and improve sperm quality in melatonin-enriched transgenic mammals (Yang et al., 2021). It can reduce oxidative stress and endoplasmic reticulum stress of mouse spermatocytes induced by heat stress, promote DSB repair of spermatocytes, and reduce apoptosis of spermatocytes (Guo et al., 2020). After melatonin administration in varicocele rat models, MDA level in testis decreased, antioxidant enzyme activity increased, and bax expression and NO levels decreased (Onur et al., 2004). Melatonin not only has the function of directly scavenging free radicals, but also has the ability to further scavenge free radicals, the stimulating products of melatonin, such as AMK and AFMK, are also powerful antioxidants. It induces the production of enzymes that convert metabolic products into harmless molecules or induce the production of other endogenous antioxidant enzymes, not only strengthens the role of antioxidant enzymes such as SOD, CAT, and GSH-Px, but also strengthens the transformation of related genes (Pablos et al., 2010). Other studies have shown that melatonin therapy in combination with varicocele surgery increased sperm parameters, peripheral blood statin B, and TAC (Lu et al., 2018). Pretreatment of human sperm with melatonin can reduce this damage by inhibiting mitochondrial ROS production, increasing mitochondrial membrane potential, decreasing the component of lipid peroxidation product 4-hydroxynonenal, and reduce sperm DNA damage and apoptosis. In conclusion, these findings suggest that melatonin is useful as a potential treatment option for infertility in men induced by heat-induced oxidative stress (Fz et al., 2021).

### Polydeoxyribonucleotide

Polydeoxyribonucleotide (PDRN) is the active part extracted from trout sperm and used for tissue repair (Altavilla et al., 2011). Stimulating adenosine A2A receptor (A2AR) can induce the production of VEGF under the pathological condition of venous blood reflux. Some studies have shown that A2AR stimulation can be an interesting target to positively modulate harmful pathophysiological signals in experimental varicocele and improve the innate mechanism of new angiogenesis by supplying oxygen and metabolites to the testis to restore spermatogenesis (Antonuccio et al., 2021). PDRN significantly increased Johnsen scores in varicocele rats and successfully improved histological damage, promoted the regeneration of new blood vessels, improve spermatogenesis, increased the content of the neuronal apoptosis inhibitory protein NAIP, and increased the survival rate of varicocele rats (Hassanin et al., 2018).

Studies show that treatment of varicocele rats with PDRN for 1 month can significantly improve testosterone levels and decreased NLRP3 inflammasome, caspase-1, and IL-1 β expression and number of TUNEL positive cells (Antonuccio et al., 2021). It improves the innate mechanism of new angiogenesis by providing compensatory oxygen and metabolites to testis, thus enhancing testis function and restoring spermatogenic function. PDRN may be a treatment option for accelerating spermatogenesis recovery after experimental varicocele (Letteria et al., 2015).

## Outlook

Varicocele, as the primary factor affecting male reproduction, is usually found by clinical palpation. Although most young varicocele cases are asymptomatic, with the increase of age and the aggravation of the course of varicocele, scrotal pain and discomfort, infertility, and testicular atrophy will slowly appear, seriously affecting the physical and mental health of patients.

Although the pathogenesis of varicocele has been widely explored, abnormal venous valve function, “Nutcracker” effect, and fluid dynamic changes caused by upright walking cannot explain the pathogenesis of varicocele. With venous blood reflux, heat stress, hypoxia, inflammation, and adrenal metabolites reflux leading to changes in the testicular microenvironment, excessive ROS is produced in pathological conditions to induce further oxidative stress and aggravate the disease. However, studies on oxidative stress in patients with varicoceles are not deep. ROS produced by stress and damage to tissues and cells after redox balance is broken can be analyzed from the perspectives of heat stress, hypoxia, and inflammation, as well as the molecular mechanism and potential pathway of damage caused by ROS from different sources to the body, and then solve some unresolved problems of varicocele.

It is an arduous and lasting task to study the role and mechanism of ROS in the generation process. There is still a long way to go to understand the impact of ROS on spermatogenesis and the mechanism leading to sterility after the local redox balance is broken. The causes of sterility caused by varicocele can be explored by studying oxidative stress related pathways. It is expected to provide treatment options and targets for infertility caused by varicocele. However, the infertility caused by varicocele and redox balance break still needs to be further studied.
